# Assessing Knowledge and Confidence in caring for Autistic Patients: An Observational Cross-Sectional Study of Maltese General Practitioners and Psychiatrists

**DOI:** 10.1192/j.eurpsy.2025.741

**Published:** 2025-08-26

**Authors:** N. Borg, E. Camilleri, L. Testa, A. Grech

**Affiliations:** 1 Mental Health Services, Attard; 2 University of Malta, Msida, Malta

## Abstract

**Introduction:**

Autism is a neurodevelopmental condition affecting 1 in 36 people worldwide. Approximately one third of autistic individuals report a diagnosed mental health condition. Autistic people constitute 20% of referrals to outpatient psychiatry clinics. Limited knowledge and awareness of autism is the main barrier to receiving appropriate diagnostic and therapeutic support. Autistic individuals’ increased mental health needs coupled with greater barriers to accessing healthcare necessitate that both primary care physicians as well as specialised services are well versed in working with these patients.

**Objectives:**

This study aimed to evaluate knowledge of autism and self-reported confidence in caring for autistic patients amongst psychiatrists and general practitioners (GPs) in Malta. This will facilitate identification of lacunae in doctors’ knowledge and advocating for greater training and awareness.

**Methods:**

A online, anonymous questionnaire was distributed amongst psychiatrists and general practitioners in Malta as well as psychiatry and GP trainees. The questionnaire consisted of demographic questions, a 22-item modified Knowledge of Autism Scale as well as a 14-item Self-efficacy Scale targeting confidence in working with autistic patients. Data was analysed using SPSS and scores were adjusted for chance responses. Scores of the psychiatry group and the GP group were compared.

**Results:**

The questionnaire was answered by 98 participants of which 60% (n=59) were female. The mean score on the knowledge of autism scale was 89.2% (SD=7.7) for psychiatry and 83.1% (SD=8.5) for GP. Mann Whitney U test revealed that psychiatrists fared better than GPs with an effect size of 0.35 (p=0.0003). The mean self-efficacy score was 6.7 (SD=0.4) for psychiatry and 5.7 (SD=0.8) for GP. Independent sample t-test revealed that psychiatrists scored better than GP (p=0.0003, 95% CI [0.509,1.49]). In both groups, there was no significant correlation between knowledge of autism scales and self-efficacy scales (psychiatry p = 0.26; GP p = 0.14).

**Conclusions:**

General practitioners, psychiatrists and their trainees overall have good knowledge about autism, and feel moderately confident in working with autistic patients. As expected, psychiatrists scored better than GPs for both knowledge and self-reported efficacy. The scores obtained are comparable to studies done on a similar population in the United Kingdom. Unfortunately, Malta stilll lacks autism diagnostic services in the public sector. Nonetheless, undergraduate medical education and postgraduate training must strive to prepare doctors for working with this common cohort of patients. This will ensure a high standard of care and avoidance of adverse health outcomes or iatrogenic harm.

**Disclosure of Interest:**

None Declared

